# lncRNA MALAT1 promotes HCC metastasis through the peripheral vascular infiltration via miRNA-613: a primary study using contrast ultrasound

**DOI:** 10.1186/s12957-022-02655-6

**Published:** 2022-06-16

**Authors:** Dandan Zhou, Ying Wang, Haifeng Hu, Huilin Liu, Jiajia Deng, Lu Li, Chunlei Zheng

**Affiliations:** 1 Department of Ultrasound, the Second Affiliated Hospital of Qiqihar Medical College, Qiqihar, 161006 China; 2 Department of Magnetic Resonance Imaging, the Second Affiliated Hospital of Qiqihar Medical College, Qiqihar, 161006 China; 3 Department of Oncology, the Second Affiliated Hospital of Qiqihar Medical College, Qiqihar, 161006 China

**Keywords:** Hepatocellular carcinoma, Metastasis, lncRNA MALAT1, miRNA-613

## Abstract

**Background:**

This study aimed to explore the specific pathogenesis of lncRNA MALAT1 promoting the invasion and metastasis of hepatocellular carcinoma (HCC) through peripheral blood vessels by regulating the expression of miRNA-613 molecule.

**Methods:**

The data of 60 HCC metastatic patients and 60 HCC non-metastatic patients detected by the contrast-enhanced ultrasound (CEUS) in the Second Affiliated Hospital of Qiqihar Medical College from January 2020 to June 2021 were collected, as well as postoperatively retained HCC tissues and paired paracancer tissues (5 cm laterally from the edge of the cancer area), to study the changes of microangiogenesis in HCC tissues with CEUS. The correlation between CEUS grading and lncRNA MALAT1 in patients with HCC was analyzed through Pearson correlation analysis, lncRNA MALAT1 and miRNA-613 in HCC tissues of patients with HCC were detected by qRT-PCR, followed by the bioinformatic analysis for the relationship between lncRNA MALAT1 and miRNA-613. The Log-growing human HCC cell strain, HepG2, was selected for experiments. Adenovirus transfection knocked down lncRNA MALAT1 in HCC cells, which was divided into two groups (inhibitor-NC group and lncR-inhibitor group), followed by knocking down miRNA-613 on the basis of knocking down lncRNA MALAT1, which was divided into three groups (inhibitor-NC group, lncR-inhibitor groups, and lncR/miR613-inhibitor group). The expression of miRNA-613 and lncRNA MALAT1 in each group was detected by qRT-PCR. The migration and invasiveness of cells in each group were detected by Transwell assay.

**Results:**

CEUS of HCC and Pearson correlation analysis showed that CEUS grading and lncRNA MALAT1 were positively correlated in patients with HCC. In HCC tissues of patients with HCC, lncRNA MALAT1 expressed high and miRNA-613 expressed low. The results of bioinformatic analysis showed the targeting of lncRNA MALAT1 and miRNA-613. Knocking down lncRNA MALAT1 could increase miRNA-613 expression significantly, and reduce the migration of HCC cells. Inhibiting miRNA-613 based on knocking down lncRNA MALAT1 could increase the survival and migration of HCC cells.

**Conclusions:**

lncRNA MALAT1 can promote HCC metastasis through the peripheral vascular infiltration by inhibiting the level of MiRNA-613, which can, therefore, be used as a potential target for the treatment of HCC.

## Highlights

1. Contrast-enhanced ultrasound (CEUS) grading was positively correlated with lncRNA MALAT1 in patients with hepatocellular carcinoma (HCC).

2. lncRNA MALAT1 expressed high and miRNA-613 expressed low in HCC tissues of patients with HCC.

3. lncRNA MALAT1 was targeted with miRNA-613.

4. Knocking down lncRNA MALAT1 could significantly increase miRNA-613 expression.

5. Knocking down lncRNA MALAT1 could reduce the migration of HCC cells.

6. Inhibiting miRNA-613 on the basis of knocking down lncRNA MALAT1 could increase the survival and migration of HCC cells.

## Background

Hepatocellular carcinoma (HCC) is the sixth most common tumor in the world [1–3], with the incidence rate increasing annually. It is also one of the most dangerous human malignancies, with more than 600,000 deaths from HCC worldwide, and the third leading cause for cancer-related deaths worldwide [4, 5]. Although China has seen a significant decrease in incidence and mortality of HCC, the huge population base and its rapid growth still lead to a large and increasing number of new HCC cases [5].

HCC is mainly correlated with viral infection, alcohol abuse, metabolic syndrome and exposure to dietary toxins such as aflatoxin and aristolochic acid [6]. It can cause extensive intrahepatic or extrahepatic metastasis through peripheral blood vessels in the very early stage of the disease. It is an invasive disease with extremely poor prognosis [4], which seriously affecting the survival rate and quality of survival of patients. Currently, although surgical resection is a first-line treatment for HCC, most patients with HCC are not suitable for surgical resection or liver transplantation because they are in an advanced stage of diagnosis with regional diffusion and metastasis. Moreover, postoperative recurrence and metastasis are quite common, ultimately causing the death of almost all patients [7]. Therefore, it is urgent to investigate the potential mechanisms of HCC metastasis through the peripheral vascular infiltration, to provide opportunities for improving clinical outcomes and developing better treatment regimens.

lncRNA is a non-coding RNA transcript, with a length that is greater than 200 nt, which can regulate gene expression at multiple levels [8]. Numerous studies have shown that lncRNA participates in multiple processes of HCC tumogenesis and development [9–11]. lncRNA MALAT1 (metastasis-correlated lung adenocarcinoma transcript 1) is a highly conserved nuclear-localized lncRNA transcribed from human chromosome 11q13, which is abundant in normal tissues [12–14]. Ping Ji et al. analyzed the expression of MALAT1 from 23 cDNAs from different healthy human organs by real-time quantitative RT-PCR. MALAT1 gene expression levels were normalized with GAPDH expression. MALAT1 expression levels were highest in pancreas and lung, and were moderate in prostate, ovary, colon, placenta, spleen, small intestine, kidney, heart, liver, testis, and brain. There was no MALAT1 expression in skin, stomach, bone marrow, and uterus [15]. It usually over-expresses in patients’ tumors and metastases, and the over-expression of lncRNA MALAT1 has been shown to be positively correlated with tumor progression and metastasis in a large number of tumor types, including breast tumors [16, 17]. It has been shown that lncRNA MALAT is prone to copy number changes in several cancer types [18, 19]. lncRNA MALAT1 has been shown to be highly expressed in many other human cancers, including but not limited to, lung cancer, breast cancer, ovarian cancer, prostatic cancer, cervical carcinoma, endometrial cancer, gastric cancer, pancreatic cancer, sarcoma, colorectal cancer, bladder cancer, cerebral cancer, hepatocellular carcinoma, esophageal squamous cell carcinoma, renal cell carcinoma, multiple myeloma, and lymphoma [20].

microRNA (miRNA) is a class of small-molecule single-stranded non-coding RNA, which is widely involved in a variety of biological processes, and closely related to tumors, diabetes mellitus and cardiovascular diseases. A large number of studies have shown that a variety of small-molecule non-coding RNAs (miRNAs) negatively regulate gene expression participating in the occurrence and development of HCC through interacting with the 3′-terminal untranslated region (3′UTR region) targeting mRNA. lncRNA MALAT1 aggravates the progression of non-small cell lung cancer by stimulating COMMD8 expression by targeting miRNA-613, and the tumorigenicity of MALAT1 has been verified in vivo [21]. miRNA-613 inhibits the expansion of HCC stem cells by modulating the SOX9 pathway. HCC stem cells promote the recurrence of HCC. However, the potential mechanism of migration of HCC stem cells remains unclear [22].

Thereout, it is hypothesized that lncRNA MALAT1 can promote HCC metastasis through the peripheral vascular infiltration by inhibiting miRNA-613 expression in HCC cells.

## Experimental methods

### Research subjects

The data of 60 HCC metastatic patients and 60 and HCC non-metastatic patients detected by the contrast-enhanced ultrasound (CEUS) were collected, see Table 1. They were admitted to the Second Affiliated Hospital of Qiqihar Medical College from January 2020 to June 2021. HCC tissues reserved after HCC surgery and paired paracancer tissues (5 cm laterally from the edge of the cancer area) were also collected. All tissues were immediately immersed in liquid nitrogen and then stored at – 80 °C until the RNA was extracted.

**Table 1:** Information of patients with liver cancer

	HCC (cases)	Non-metastatic (cases)	Metastatic (cases)
**Total**	60 (*n* = 60)	22 (*n* = 60, 36.67%)	38 (*n* = 60, 63.33%)
**Male**	30 (*n* = 60, 50%)	10 (*n* = 30, 33.33%)	20 (*n* = 30, 66.67%)
**Female**	30 (*n* = 60, 50%)	12 (*n* = 30, 40%)	18 (*n* = 30, 60%)
**Age (years)**	30–70	30–70	30–70

#### Inclusion criteria

Patients who voluntarily signed the informed consent; Patients who met the 2010 Diagnostic Guidelines for HCC issued by AASLD; and Patients without other malignant tumor diseases. Among them, 30 males and 30 females aged 30~70 years old were randomly selected.

#### Exclusion criteria

Patients with consciousness or communication disorders; patients who have been treated with other malignant tumor diseases; patients unwilling to cooperate with the study arrangement; and patients with undetected nodules will not be included in the study temporarily.

All individuals provided informed written consent to use their tissues in this experimental study, which was approved by the Ethics Committee of the Second Affiliated Hospital of Qiqihar Medical College, with the research method that met the criteria set by the Declaration of Helsinki.

Microangiogenesis of HCC tissues was observed by CEUS, with unchanged parameters (mechanical indexes, gains and depths). The tumor sizes and blood flow signal were recorded, and graded as 0, 1, 2, and 3 with numbers from low to high. Grade 0 meant the tumor tissue with no blood flow signal or blood flow activity. Grade 1 meant the tumor tissue with short star-shaped blood flow signals, which were less distributed, appear and disappear occasionally. Grade 2 meant the tumor tissue with continuous linear blood flow signals and continuous blood flow activity. Grade 3 meant the tumor tissue with larger vessels passing through and fast blood flow.

### Cell lines and cell cultures

The log-growing human HCC cell strains, HepG2 and Hep3B, were selected for experiments. The cell line was purchased from BMCR. HepG2 and Hep3B cells were cultured in DMEM (Hyclone, USA) in 10% FBS (Thermo Fisher Scientific, USA), 100 U/ml penicillin/streptomycin (Sigma) and 5% CO_2_ at 37°C.

### lncRNA MALAT1 and miRNA-613 in HCC tissues and paracancer cells of patients with HCC were detected by qRT-PCR

Total RNA of tumor tissues and paired paracancer tissues were extracted from HCC metastatic patients and HCC non-metastatic patients with the RNeasy mini kit, after which cDNA was synthesized by reverse transcription (RT) with the Primescript RT kit (Takara, China), and the expression of lncRNA MALAT1 and miRNA-613 was detected by PCR amplification. The upstream primer of lncRNA MALAT1 was 5′-GCTCTGTGGTGTGGGATTGA-3′, the anti-sense of the downstream primer was 5′-GTGGCAAAATGGCGGACTTT-3′. The upstream primer of miRNA-613 was 5′-GTGAGTGCGTTTCAAGTGT-3′, and the downstream primer was 5′-TGAGTGGCAAAGAAGGAACAT-3′. The upstream primer of U6 was 5′-CTCGCTTCGGCAGCACA-3′, and the downstream primer was 5′-AACGCTTCACGAATTTGCGT-3′.

PCR amplification was performed with the 25 μl reaction system at 95 °C for 30 s, with predenaturation of 1 cycle, at 95 °C for 5 s, with denaturation of 40 cycles, and at 60 °C for 20 s, with annealing for 1 cycle. Based on the obtained Ct value, the relative RNA was expressed as 2^−ΔΔCt^. All the experiments were repeated three times.

### Construction of vectors and adenovirus transfection

The log-growing human HCC cell strains, HepG2 and Hep3B, were selected. lncRNA MALAT1 was knocked down in HepG2 and Hep3B cells. Short hairpin RNA for lncRNA MALAT1 was designed and synthesized, and cloned into pSicoR plasmid vector (Genepharma Co., Ltd., Shanghai, China). The subcultured cells were inoculated in 6-well plates and cultured until 70% of the cultured cells converged, which were divided into two groups (inhibitor NC group and lncR-inhibitor group). Fifty microliters vector with the titer of 3.45 × 10^10^ PFU/ml was added to each group. After 48 hours of culture, lncRNA MALAT1 expression was detected by qRT-PCR to verify the transfection efficiency.

Thereafter, miRNA-613 inhibitor was added on the basis of knocking down lncRNA MALAT1, and the cells were divided into three groups (inhibitor-NC group, lncR-inhibitor group and lncR/miR613-inhibitor group). Twenty microliters of miRNA-613 inhibitor (1 μg) was added to each group, and after 48 h of culture, miRNA-613 expression was detected with qRT-PCR to verify the transfection efficiency. After another 48 h of transfection, the cells were gained and used in in vitro experiments.

### Transwell experiments

The invasiveness of cells was detected among inhibitor-NC and lncR-inhibitor groups and inhibitor-NC, lncR-inhibitor, and lncR/miR613-inhibitor groups. To assess the invasiveness of the cells, transfected HCC cells were inoculated on the upper chamber surface of the bottom membrane of Matrigel-coated Transwell chamber containing 500 μl of serum-free DMEM medium. The lower chamber was filled with 500 μl of DMEM medium containing 10%FBS. After 24 h of incubation in a humidified room at 37 °C, cells on the surface of the lower membrane were fixed with 100% methanol for 30 min, followed by crystal violet staining for 20 min. Invasive cells were photographed and counted with the inverted microscope (Leica Microsystems, Tokyo, Japan). All of the experiments were repeated three times.

### Target prediction

Several families of miRNA-613 were predicted with the software Human microRNA families. The location, conservation, and base sequence of miRNA-613 in human genome were obtained with the online target gene prediction software, UCSE Genome Brower and miRbase database. The software, TargetScan, miRanda, and MicroT-CD, were selected to predict the target genes of miRNA-613, and their intersection was taken. Then, the miRTarBase database was used to find the target genes verified by experiments. The results were combined with the previous intersection as the target gene set for further analysis. GeneOntology and DAVID database were used for biological function enrichment analysis (GO analysis) and pathway analysis on the predicted target gene set of miRNA-613. Fisher’s exact test and *R*^2^ test were used to obtain statistically significant signal transduction pathways and disease pathways with *P* < 0.05 as the significance threshold.

### Luciferase reporter assay

The expression vectors of wild-type miRNA-613 3′UTR and mutant miRNA-613 3′UTR were successfully constructed first, which was operated according to the instructions of the luciferase detection kit (Sangon Biotech, Shanghai), and the detection results were observed after 48 h with the double luciferase reporter analysis system (PROMEGA) and counted.

### Statistical analysis

The data were shown as the mean ± SD (standard deviation) of three independent experiments, and the statistical analysis was processed with the SPSS 24.0 statistical software (SPSS Inc. Chicago, IL, USA). *t* test was adopted for the comparison between the two groups, ANOVA was adopted for the difference between the two groups, and Pearson correlation analysis was applied to analyze the correlation between CEUS grading and lncRNA MALAT1 in patients with HCC. *P* value was calculated bilaterally. SPSS 24.0 software was used to perform ROC curve analysis on the qPCR data of lncRNA MALAT1 and miRNA-613 and CEUS liver cancer diagnosis. *P* < 0.05 was defined as statistically significant.

## Results

### CEUS grading of patients with HCC

In order to explore the correlation between CEUS grading and lncRNA MALAT1 in patients with HCC, the data of HCC metastatic patients and HCC non-metastatic patients detected by CEUS in the past one and a half years were collected, and the changes in microangiogenesis of HCC tissues were observed by CEUS.

**Fig. 1:**
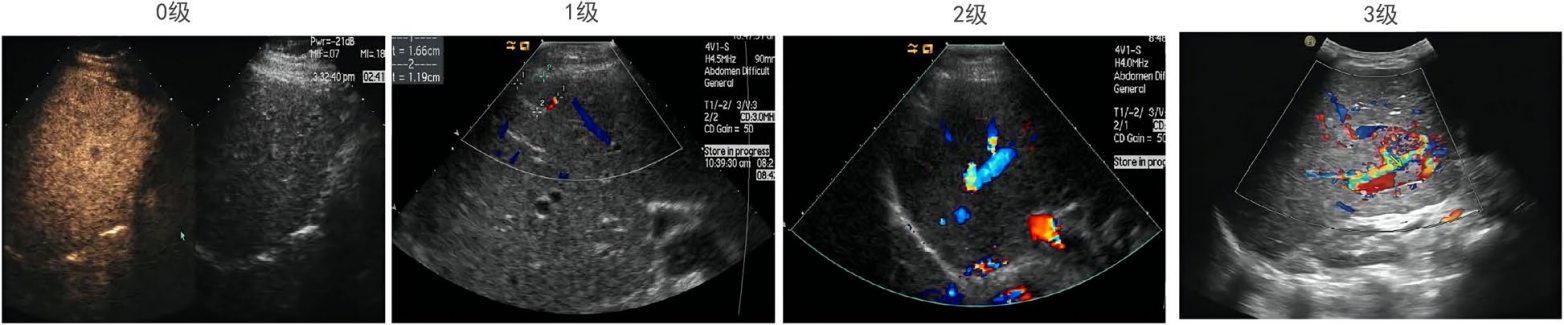
CEUS grading of patients with HCC

Patients with HCC were grouped according to different CEUS grading. The tumor sizes and blood flow signal were detected, and graded as 0, 1, 2, and 3 with numbers from low to high. Grade 0 meant the tumor tissue with no blood flow signal or blood flow activity. Grade 1 meant the tumor tissue with short star-shaped blood flow signals, which were less distributed, appear and disappear occasionally. Grade 2 meant the tumor tissue with continuous linear blood flow signals and continuous blood flow activity. Grade 3 which meant the tumor tissue with larger vessels passing through and fast blood flow (Fig. 1).

### Prediction of the target relationship between lncRNA MALAT1 and miRNA-613

**Fig. 2:**
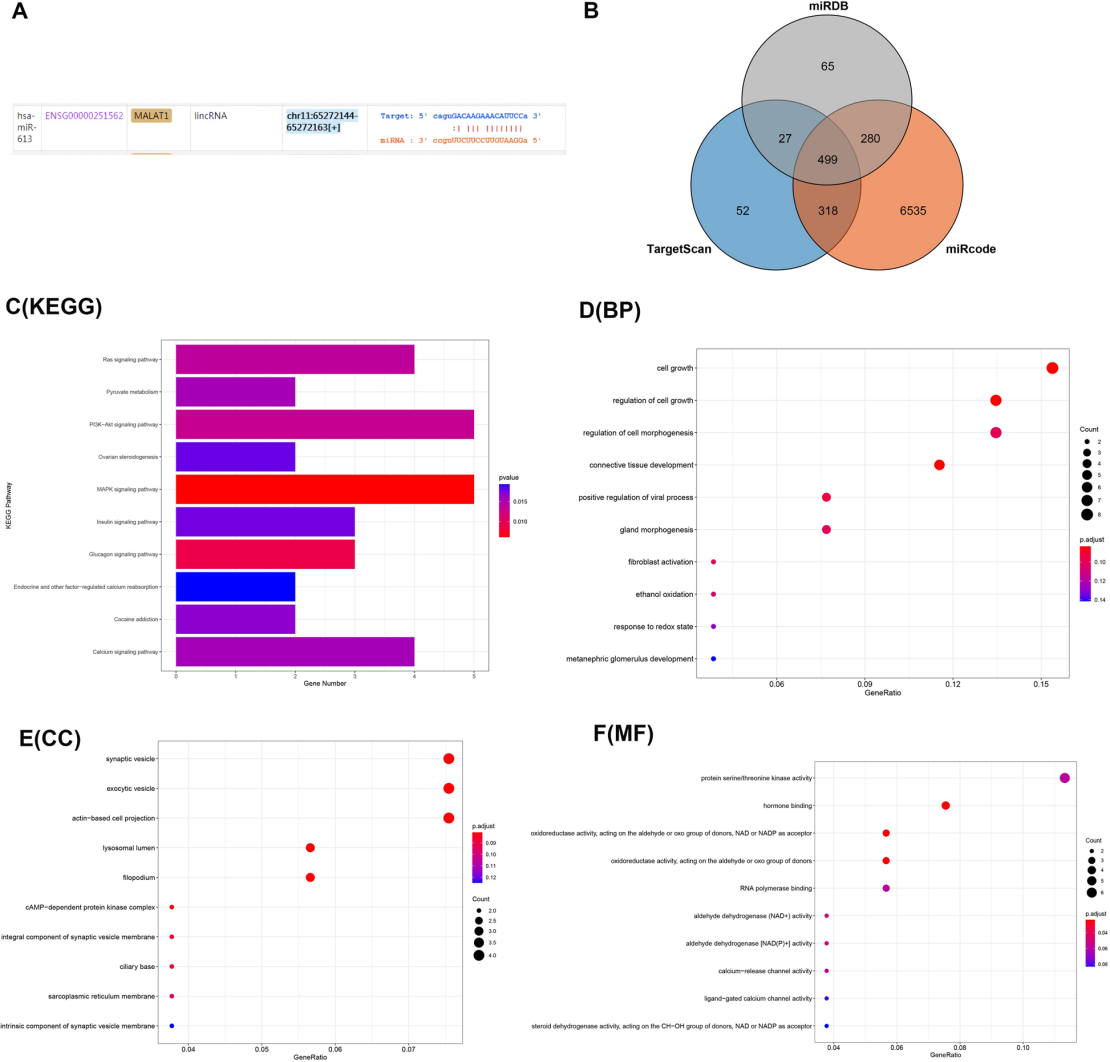
Prediction of targeting relationship between lncRNA MALAT1 and miRNA-613. **A** ENCORII and miRcode webpage prediction tools were used to confirm the mutual relationship. **B** TargetScan, miRcode, and miRDB webpage prediction tools were used to predict the target genes of miRNA-613, and the intersection were taken to get 499 target genes. **C**–**F** Biological function enrichment analysis (GO analysis) and pathway analysis were performed on the set of predicted target genes of miRNA-613 by R software. *P* < 0.05 was significant

In order to clarify the relationship between lncRNA MALAT1 and miRNA-613 in both HCC metastatic patients and HCC non-metastatic patients, several families of miRNA-613 were predicted with the software Human microRNA families. ENCORII and miRcode web prediction tools were used to confirm the interaction between the two factors (Fig. 2A). The location, conservation, and base sequence of miRNA-613 in human genome were obtained with the online target gene prediction software, UCSE Genome Brower and miRbase database. The software, TargetScan, miRcode, and miRDB Web Page Prediction Tool were selected to predict the target genes of miRNA-613, their intersection was taken, 499 target genes were obtained (Fig. 2B). GeneOntology and DAVID database were used for biological function enrichment analysis (GO analysis) and pathway analysis on the predicted target gene set of miRNA-613 using R software (Fig. 2C–F). Fisher’s exact test and *R*^2^ test were used to obtain statistically significant signal transduction pathways and disease pathways with *P* < 0.05 as the significance threshold. The results showed that lncRNA MALAT1 targets the regulation of miRNA-613 in HCC tissues of HCC metastatic patients.

### lncRNA MALAT1 expressed high and miRNA-613 expressed low in HCC tissues of HCC metastatic patients

**Fig. 3:**
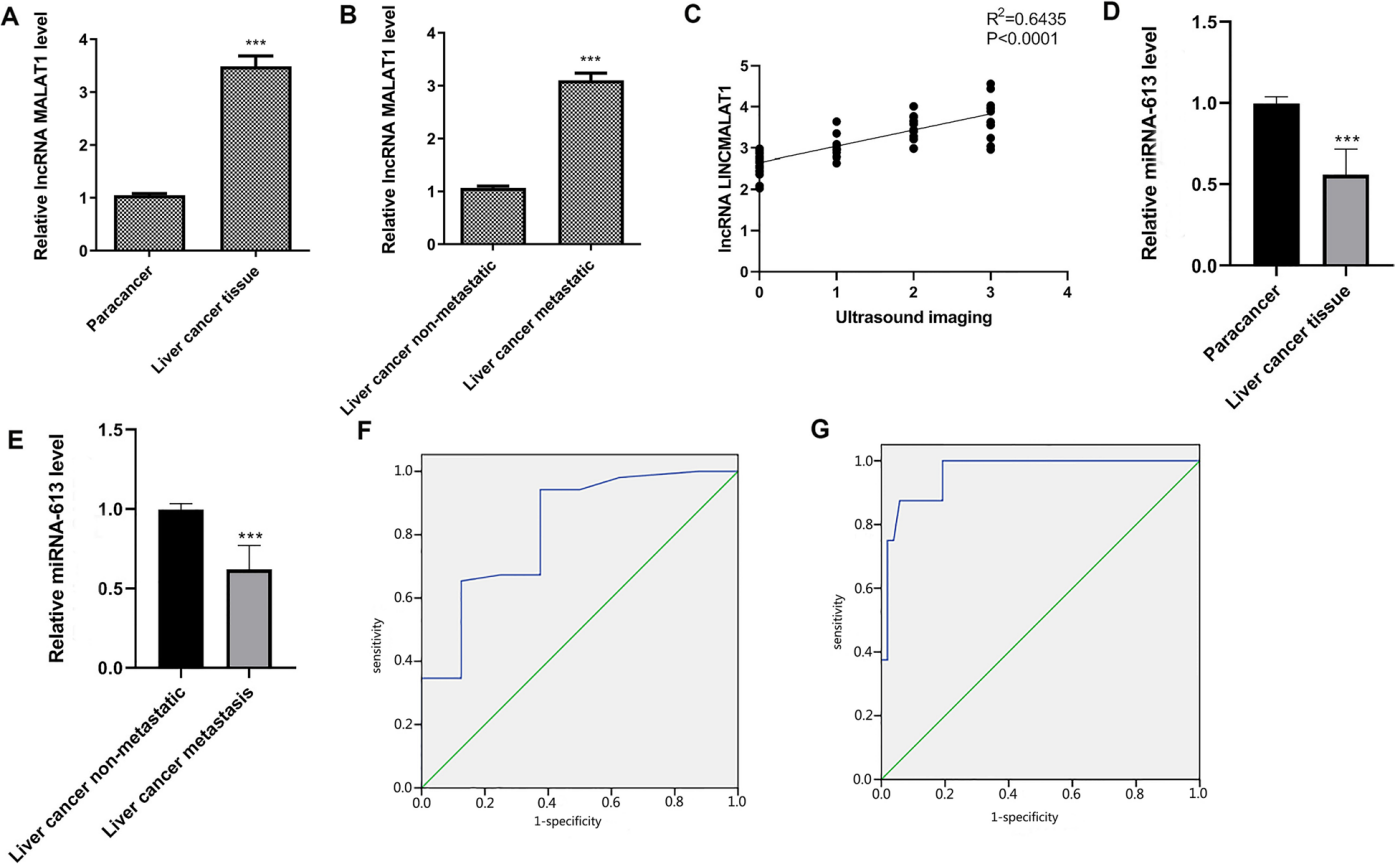
lncRNA MALAT1 expressed high and miRNA-613 expressed low in HCC metastatic patients. **A** lncRNA MALAT1 expression in HCC tissues and paracancer tissues of patients with HCC was detected by qRT-PCR, ^***^P < 0.001 vs paracancer, *n* = 120. **B** lncRNA MALAT1 expression in HCC tissues of HCC metastatic patients and HCC non-metastatic patients was detected by qRT-PCR, ^***^P < 0.001 vs HCC non-metastatic, *n* = 60. **C** CEUS grading and lncRNA MALAT1 in patients with HCC were analyzed through Pearson correlation analysis, *n* = 60. **D** miRNA-613 expression in HCC tissues and paracancer tissues of patients with HCC was detected by qRT-PCR, ^***^P < 0.001 vs paracancer, *n* = 120. **E** miRNA-613 expression in HCC tissues of HCC metastatic patients and HCC non-metastatic patients was detected by qRT-PCR was detected by qRT-PCR. **F** SPSS 24.0 software was used for ROC curve analysis of lncRNA MALAT1 and CEUS liver cancer diagnosis. **G** SPSS 24.0 software was used for ROC curve analysis on miRNA-613 and CEUS liver cancer diagnosis. ^***^P < 0.001 vs HCC non-metastatic, *n* = 60

HCC tissues and paired paracancer tissues of patients were taken for grinding, total RNA was also extracted from the tissues. The level of lncRNA MALAT1 in HCC tissues and paired paracancer tissues of different grades of patients with HCC were detected by qRT-PCR. The result showed that compared with paired paracancer tissues, lncRNA MALAT1 expression increased in HCC tissues in patients with HCC (Fig. 3A, *P* < 0.001), and compared with the HCC non-metastasis group, lncRNA MALAT1 expression increased in HCC tissues in HCC metastatic patients (Fig. 3B, *P* < 0.001), suggesting that lncRNA MALAT1 expresses high in the HCC tissues of HCC metastatic patients.

Then, the correlation between CEUS grading and lncRNA MALAT1 in patients with HCC was analyzed according to the Pearson correlation analysis. The results showed that CEUS grading of HCC metastatic patients was positively correlated with the lncRNA MALAT1 expression in HCC tissues of HCC metastatic patients (Fig. 3C). The above results suggest that lncRNA MALAT1 may have some correlation with HCC metastasis through peripheral vascular infiltration in patients with HCC.

Besides, miRNA-613 expression was detected with qRT-PCR, showing that miRNA-613 expression decreased in HCC tissues of patients with HCC compared with paracancer tissues (Fig. 3D, *P* <0.001), and decreased in HCC tissues of HCC metastatic patients compared with those of HCC non-metastatic patients (Fig. 3E, *P* < 0.001), which suggested that miRNA-613 is negatively correlated with the metastasis of HCC cells through peripheral vascular infiltration in patients with HCC.

SPSS 24.0 software was used to perform ROC curve analysis on lncRNA MALAT1 and CEUS for the diagnosis of liver cancer. The area under the curve was 0.821, *P* = 0.004, which was statistically significant, indicating that lncRNA MALAT1 was significant in the diagnosis of liver cancer. SPSS 24.0 software was used to perform ROC curve analysis on miRNA-613 and CEUS for the diagnosis of liver cancer. The area under the curve was 0.963, *P* < 0.001, which was statistically significant, indicating that miRNA-613 was significant for the diagnosis of liver cancer.

### Knocking down lncRNA MALAT1 could significantly increase miRNA-613 expression

**Fig. 4:**
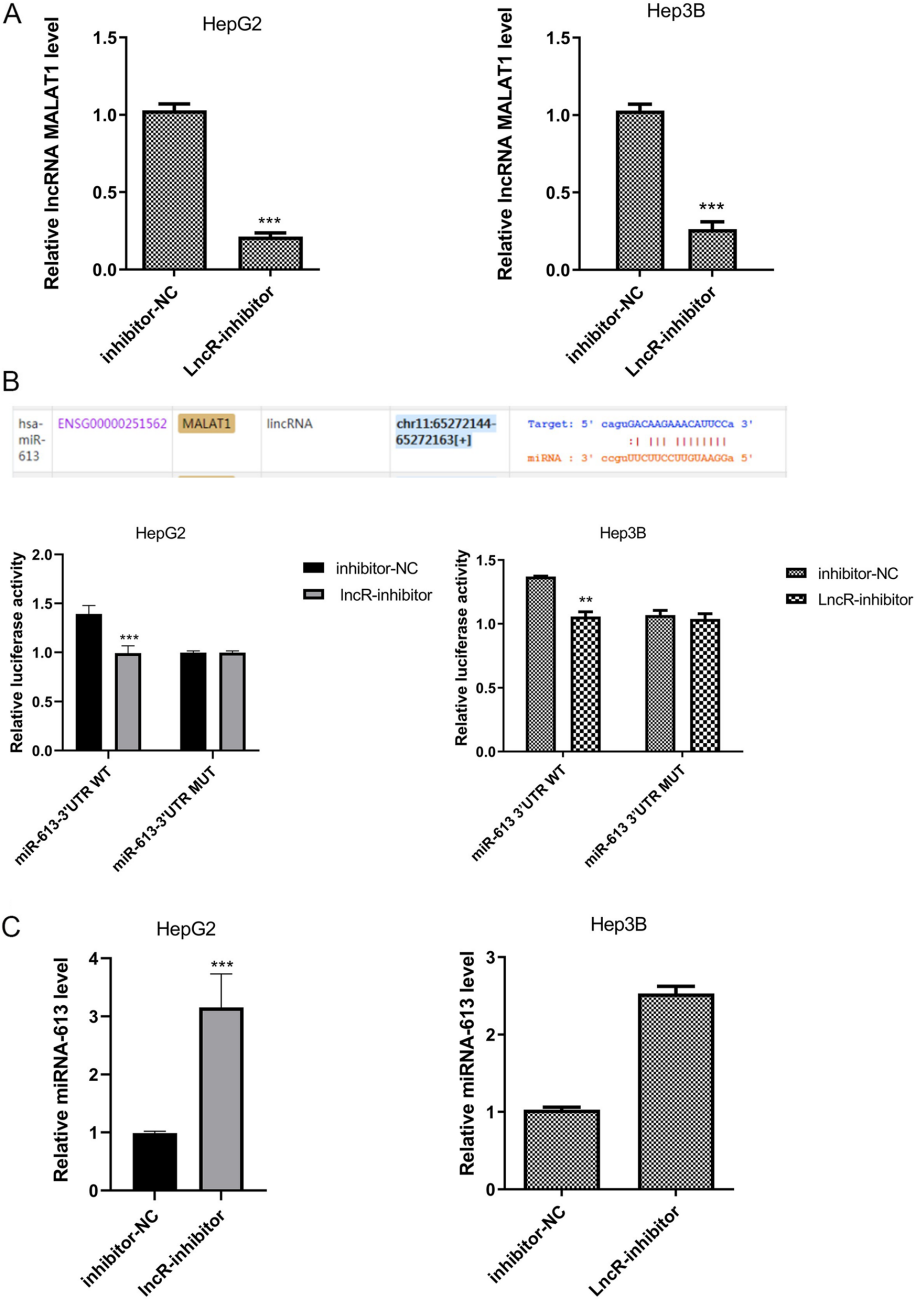
Knocking down lncRNA MALAT1 could significantly increase miRNA-613 expression. **A** lncRNA MALAT1 expression in HCC cells was detected by qRT-PCR, ^***^P < 0.001 vs inhibitor-NC, *n* = 10. **B** Luciferase reporter assay, ^***^P < 0.01 vs miRNA-613 3′UTR WT, *n* = 6. **C** miRNA-613 expression in HCC cells was detected by qRT-PCR, ^***^P < 0.001 vs inhibitor-NC, *n* = 10

The log-growing human HCC cell strain, HepG2 and Hep3B, were selected. lncRNA MALAT1 was knocked down in HepG2 and Hep3B cells. lncRNA MALAT1 expression in HCC cells was detected by qRT-PCR, showing that lncRNA MALAT1 expression decreased in the lncR-inhibitor group compared with the inhibitor-NC group (Fig. 4A, *P* < 0.001), indicating the success of the construction of the lncRNA MALAT1 silencing model.

A miRNA-613 wild-type and mutant double-luciferase reporter system was constructed to verify the hypothesis. The luciferase reporter assay showed that lncR-inhibitor could improve the wild-type miRNA-613 3′UTR luciferase activity, and had no effect on the mutant miRNA-613 3′UTR luciferase activity (Fig. 4B, *P* < 0.001), suggesting that miRNA-613 3′UTR has a site binding to lncRNA MALAT1.

To further clarify the targeting of lncRNA MALAT1 and miRNA-613 in HCC cells, miRNA-613 expression in HCC cells was detected by qRT-PCR, showing that miRNA-613 expression was significantly higher in the lncR-inhibitor group compared with the inhibitor-NC group (Fig. 4C, *P* < 0.001), which suggest that lncRNA MALAT1 inhibits miRNA-613 expression in HCC cells.

### Knocking down lncRNA MALAT1 could reduce the migration of HCC cells

**Fig. 5:**
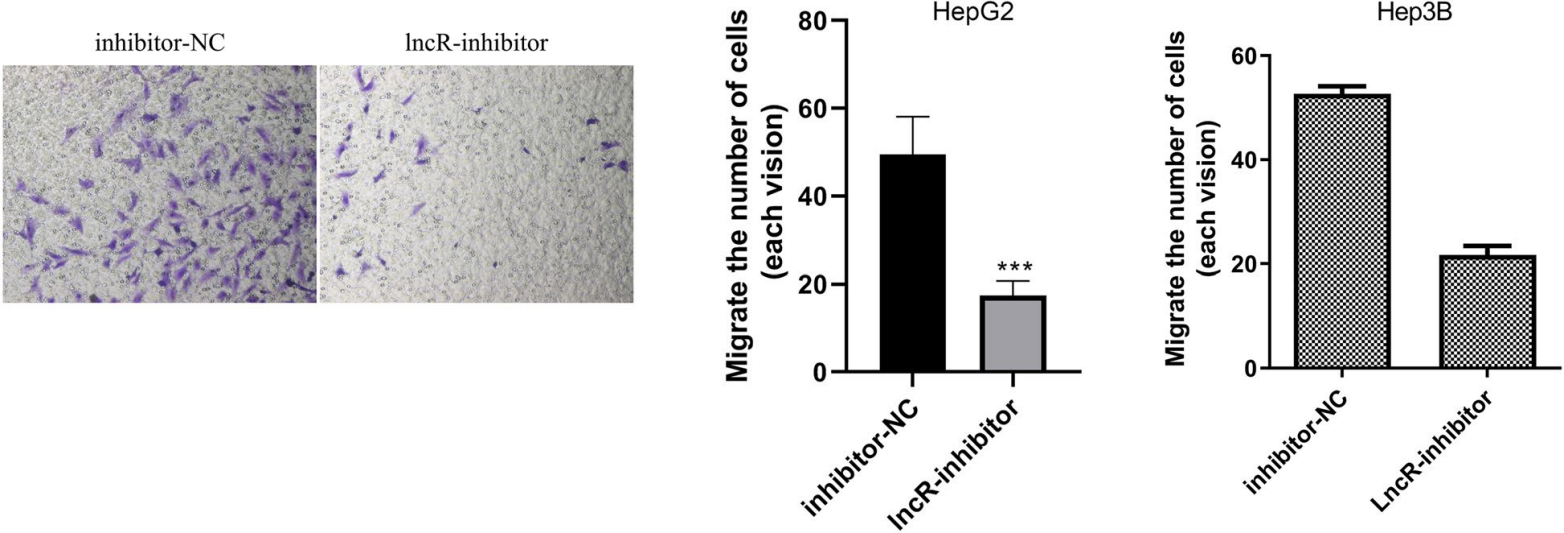
Knocking down lncRNA MALAT1 could reduce the migration of HCC cells. Note: The invasiveness expression of HCC cells was detected by the Transwell assay, ^***^P < 0.001 vs inhibitor-NC, *n* = 10

To further explore the correlation between the metastasis of HCC cells through peripheral vascular infiltration with lncRNA MALAT1, the invasiveness of HCC cells in the inhibitor-NC and lncR-inhibitor groups was detected with the Transwell assay. The results showed that the invasiveness of HCC cells in the lncR-inhibitor group was weaker than that of the inhibitor-NC group (Fig. 5, *P* < 0.001), suggesting that lncRNA MALAT1 can promote the metastasis of HCC cells through peripheral vascular infiltration.

### Inhibiting miRNA-613 on the basis of knocking down lncRNA MALAT1 could increase the migration of HCC cells

**Fig. 6:**
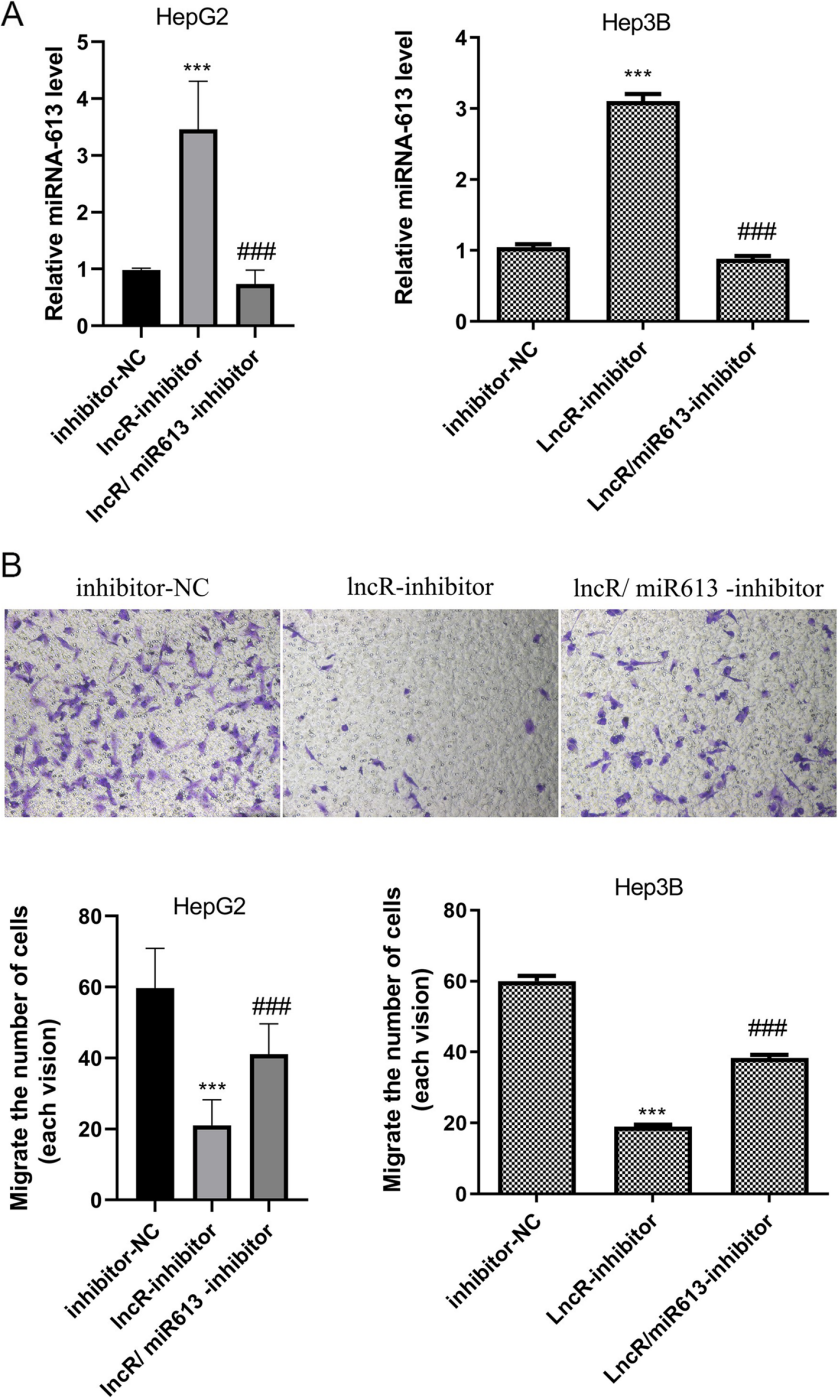
Inhibiting miRNA-613 on the basis of knocking down lncRNA MALAT1 could increase the migration of HCC cells. Note: **A** miRNA-613 expression in HCC cells was detected by qRT-PCR, ^***^P < 0.001 vs inhibitor-NC, ^*###*^*P* < 0.001 vs lncR-inhibitor, *n* = 10. **B** The invasiveness expression of HCC cells was detected by the Transwell assay, ^***^P < 0.001 vs inhibitor-NC, ^*###*^*P* < 0.001 vs lncR-inhibitor, *n* = 10

To explore whether lncRNA MALAT1 can promote the metastasis of HCC through peripheral vascular infiltration by inhibiting miRNA-613 expression in HCC cells, miRNA-613 inhibitor was added on the basis of knocking down lncRNA MALAT1, and the cells were divided into three groups (including inhibitor-NC, lncR-inhibitor, and lncR/miR613-inhibitor groups) and miRNA-613 expression in HCC cells was detected by qRT-PCR. miRNA-613 expression increased in the lncR-inhibitor group compared with the inhibitor-NC group (Fig. 6A, *P* < 0.001) and decreased in the lncR/miR613-inhibitor group compared with the lncR-inhibitor group (Fig. 5A, *P* < 0.001), indicating the success of lncR LINC MALAT1/miR613 inhibition.

The invasiveness of HCC cells in the three groups was detected with the Transwell assay. The results showed that the invasiveness of HCC cells decreased in the lncR-inhibitor group compared with the inhibitor-NC group (Fig. 6B, *P* < 0.001), and that of HCC cells in the lncR/miR613-inhibitor group increased compared with that of the lncR-inhibitor group (Fig. 6B, *P* < 0.001), suggesting that lncRNA MALAT1 can promote the metastasis of HCC through peripheral vascular infiltration by inhibiting miRNA-613 expression in HCC cells.

## Discussion

In recent years, the in-depth study on the pathogenesis and effective treatment of HCC have become the focus of research. Nowadays, long-chain non-coding RNA has become a new focus in the research on cancers. lncRNA do not encode protein capabilities, but have a significant role in epigenetics and regulating gene expression, as well as in various cancers. As ceRNA, lncRNA binds to miRNA, affecting the silencing effect of miRNA on mRNA, and indirectly regulating the expression of mRNA molecules, thereby affecting cell proliferation, migration and invasion [23, 24]. HCC is a highly vascularized solid tumor with rapid growth and rapid metastasis through peripheral vascular invasion, as well as poor prognosis [25, 26], low survival and poor quality of survival in patients, but the specific mechanism of HCC cell metastasis through peripheral vascular infiltration in patients with HCC is not clear.

In the current study, we mainly explored the role of lncRNA MALAT1 in HCC and HCC metastasis through peripheral vascular infiltration. This study collected the data of HCC metastasis patients detected by CEUS in the past 1 year. The target relationship between lncRNA MALAT1 and miRNA-613 in HCC tissues of patients with HCC was detected through the bioinformatics analysis. lncRNA MALAT1 targeting the regulation of miRNA-613 was later verified by a double-luciferase reporter gene. The levels of lncRNA MALAT1 in HCC tissues with different grades of patients with HCC was detected by qRT-PCR. The results showed a high expression of lncRNA MALAT1 in HCC tissues of patients with HCC. Pearson correlation analysis showed that the level of contrast-enhanced ultrasound in patients with hepatocellular carcinoma was positively correlated with the expression level of lncRNA MALAT1 in liver cancer tissues of patients with hepatocellular carcinoma. lncRNA MALAT1 was knocked down in liver cancer cells, and the expression of miRNA-613 in liver cancer cells was detected by q-PCR. After knockdown, the expression of miRNA-613 in liver cancer cells was significantly increased, and the invasion ability of liver cancer cells was significantly weakened. On the basis of inhibiting miRNA-613, the invasive ability of hepatoma cells was enhanced. It is suggested that in liver cancer cells, lncRNA MALAT1 can inhibit the expression of miRNA-613 and promote the invasion and metastasis of liver cancer cells through peripheral blood vessels.

In recent years, more and more studies have been conducted on the role of lncRNA MALAT1 in tumors, and studies have shown that the expression of lncRNA MALAT1 is abnormally elevated in primary human non-small cell lung tumors with high metastatic tendency [20]. The expression of lncRNA MALAT1 is associated with ERAT1 breast tumors and lower recurrence-free survival (RFS) [27], and lncRNA MALAT1 can promote the occurrence and development of breast cancer by up-regulating the WNT/W-catenin (CTNNB1) pathway [28]. It has been observed that LINC MALAT1 has a similar promoting effect on the growth and invasion of colorectal cancer tumor cells as the liver cancer in this study, but has diametrically opposite results in the growth and invasion of glioma and breast cancer. It suggested that the mechanism of lncRNA MALAT1 in the growth and invasion of different tumor cells is different, which may be related to the microenvironment and physiological barriers of different tumors [29–32]. The study by Wei Shuquan et al. demonstrated that MALAT1 and PD-L1 are highly expressed in NSCLC and are associated with significantly down-regulated miR-200a-3p. MALAT1 and PD-L1 constitute a ceRNA regulatory network involved in NSCLC progression [33]. Yang Tian et al. demonstrated that MALAT1 plays an important role in the chemosensitivity of NSCLC. By targeting the miR-197-3p/p120 signaling axis, MALAT1 increases lung cancer cell resistance to cisplatin and cellular resistance to tetracycline, gefitinib, and paclitaxel [34]. The study by Wang Zhujuan et al found that MALAT1 was overexpressed in sunitinib-resistant renal cell carcinoma tissues and cells, indicating that knockout of MALAT1 inhibited chemotherapy resistance, cell proliferation and invasiveness, and enhanced apoptosis. MALAT1 has been shown to act as a ceRNA of miR-362-3p, and controls its downstream target G3BP1. Furthermore, the resistance of renal cancer cells to sunitinib is mediated by the MALAT1/miR-362-3p/G3BP1 signaling axis [35]. The study of Zhao Liying et al. found that the expression of MALAT1 was significantly increased in liver cancer stem cells, and this lncRNA could serve as the ceRNA of miR-375 to maintain the stemness of cancer stem cells, while regulating the expression of the downstream target gene YAP1 [36].

MicroRNAs (miRNAs) negatively regulate gene expression by interacting with the 3′-terminal untranslated region (3′UTR region) of target mRNAs and participate in the occurrence and development of HCC. The study of Zhu K et al. found that miRNA-613 is abnormally lowly expressed in breast cancer tissues and cells. miRNA-613 can specifically bind to the 3'UTR of SOX9, and miRNA-613 may inhibits the proliferation, invasion and metastasis of breast cancer cells and epithelial-mesenchymal transition by regulating SOX9 and Wnt/β-catenin signaling pathway [37]. The study by Jiang Xuemei et al. demonstrated that the 3′-untranslated region (UTR) of the miR-613 target region was expressed in YWHAZ and regulated its expression in HCC cells, and overexpression of YWHAZ could partially abolish the upregulation of tumor suppressor effect induced by miR-613 in liver cancer cells [38]. The study by Ning Zhou et al. demonstrated that the expression of miR-613 was significantly downregulated in HCC tissues and cells, and RMRP could act as a ceRNA to negatively regulate the expression of miR-613. It suggested that the RMRP/miRNA-613 axis could serve as a new molecular target for the treatment of HCC [39]. The study of Wang S et al. showed that the expression of lncRNA MALAT1 is abnormally elevated in NSCLC tissues and cells, and lncRNA MALAT1 up-regulates the level of COMMD8 by competitively targeting miRNA-613, thereby playing an oncogenic role in NSCLC [20]. These studies all implicate miRNA-613 as a new potential therapeutic target for HCC.

There are several other lncRNAs that are also potential targets for therapy in terms of tumor development and metastasis. The study by Hui Wang et al. found that HOXD-AS1 in lncRNA was significantly upregulated in HCC tissues. Clinical studies have shown that high expression of HOXD-AS1 is associated with poor prognosis and high lymph node metastasis in HCC patients, and is an independent risk factor for survival. STAT3 can specifically interact with the promoter of HOXD-AS1 to activate the transcription of HOXD-AS1. Knockdown of HOXD-AS1 significantly inhibited the migration and invasion of hepatoma cells in vitro and distant lung metastasis in vivo. Overexpression of HOXD-AS1 competitively binds miR-130a-3p and prevents SOX4 miRNA-mediated degradation, thereby activating the expression of EZH2 and MMP2, and promoting liver cancer metastasis [40]. The study by Cao HL et al. found that lncRNA RMRP was highly expressed in bladder cancer tissues compared with adjacent tissues, and also found that the expression of RMRP was closely related to the size of patients, lymph node metastasis and survival time. In addition, RMRP can promote the proliferation, migration and invasion of BC cell lines by regulating miR-206 [41]. To explore whether lncRNA MALAT1 can promote HCC metastasis through peripheral vascular infiltration by inhibiting miRNA-613 expression in HCC cells on the basis of knocking down lncRNA MALAT1, the invasiveness of HCC cells was detected with the Transwell assay again. The results showed that the invasiveness of HCC cells increased after inhibiting miRNA-613 on the basis of knocking down lncRNA MALAT1, suggesting that lncRNA MALAT1 can promote HCC metastasis through peripheral vascular infiltration by inhibiting miRNA-613 expression in HCC cells.

## Conclusions

The study is only a preliminary exploration of HCC metastasis through peripheral vascular infiltration, and the specific mechanisms still need to be clarified by further studies. Thus, in our study, we found that lncRNA MALAT1 can promote HCC metastasis through peripheral vascular infiltration by inhibiting the levels of miRNA-613, and lncRNA MALAT1 may be a potential target for the treatment of HCC.

## Data Availability

The datasets used and/or analyzed during the present study are available from the corresponding author on reasonable request.
